# Influence of Pseudowollastonite on the Performance of Low Calcium Amorphous Hydraulic Binders

**DOI:** 10.3390/ma12203457

**Published:** 2019-10-22

**Authors:** Daniela Santos, Rodrigo Lino Santos, João Pereira, Ricardo Bayão Horta, Rogério Colaço, Patrizia Paradiso

**Affiliations:** 1 Instituto Superior Técnico, University of Lisbon, Av. Rovisco Pais, 1049-001 Lisboa, Portugal; daniela6_santos@hotmail.com (D.S.); RLSantos@cimpor.com (R.L.S.); teresa.empis@cires.pt (R.B.H.); rogerio.colaco@tecnico.ulisboa.pt (R.C.); 2 CIMPOR - Cimentos de Portugal, SGPS S.A., Avenida José Malhoa 22, 1070-049 Lisboa, Portugal; JPereira@cimpor.com; 3 IDMEC, Instituto Superior Técnico, Universidade de Lisboa, Av. Rovisco Pais, 1049-001 Lisboa, Portugal

**Keywords:** hydraulic binders, decarbonation, cement, pseudowollastonite

## Abstract

A systematic investigation of the influence of pseudowollastonite on the performance of a new family of low calcium hydraulic binders is described. Samples of the new low calcium binder were produced by an innovative process consisting of heating and homogenizing the mix of raw materials (limestone, sand, and fuel cracking catalyst) at a constant temperature followed by the rapid cooling of the mixture itself. Different maximum temperatures, close to the melting temperature of the mix, were tested, and materials with CaO/SiO_2_ (C/S) ratios of 0.9, 1.1, and 1.25 were produced into the form of the amorphous phase with small percentages of pseudowollastonite. Compressive strength results were determined at 7, 28, and 90 days of hydration, and the hydrated phases were analyzed using isothermal calorimetry, X-ray diffraction (XRD) analysis, thermogravimetry analysis (TGA), scanning electron microscopy (SEM), and differential scanning calorimetry (DSC). The present work is focused on the influence of the percentage of the pseudowollastonite phase on the binder compressive strength performance.

## 1. Introduction

Portland cement (PC) is the primary component of concrete, and its production stands today between 2 and 3 billion tons per year, and it is expected to grow to 5 billion tons per year by 2050 [1]. Despite the fact that this growth represents a socio-economic positive event, its potential impact on climate change is an issue that warrants particular attention. Nowadays, the production of 1 ton of PC releases an estimated 0.73–0.99 tons of CO_2_ [2], representing 5%–6% of total man-made greenhouse gases [2]. Hence, within the scope of the 2030 United Nations (UN) agenda, developing hydraulic binders that match the technical and economic qualities of PC, but allow a reduction of the carbon footprint, is a target and a challenge both for researchers and for the cement industry. 

Being that the decarbonation reaction is the main source of CO_2_ emissions in clinker production [2], different approaches have been investigated to lower the calcium percentage in the raw mix: e.g., the substitution of calcium for other elements; the development of belitic clinkers; the development of alternative non-clinker technological routes. Representative state of the art examples are described in patents and the literature [3,4,5,6,7], but none of these solutions has yet been fully assimilated by the cement industry.

Recently our group proposed an innovative and simple new approach consisting of the amorphization of a low calcium binder, which would allow the reduction of CO_2_ emissions up to 33% [8,9,10,11,12]. The present work follows on from these previous investigations and presents two main objectives: The optimization of the ratio CaO/SiO_2_ (C/S) in the range 0.9 to 1.25, and the investigation of the effect of the pseudowollastonite phase on the compressive strength performance.

## 2. Materials and Methods 

Three types of amorphous binders, with a C/S ratio, respectively, of 0.9, 1.1, and 1.25, were produced by milling and mixing the raw materials (limestone, sand, and fuel cracking catalyst (FCC), the latter as a source of Al_2_O_3_). The raw materials were ground to a particle size below 200 μm in a ringmill, and the chemical composition was obtained through wavelength-dispersive X-ray fluorescence spectrometry (WD-XRF), using an Axios cement, (Malvern Panalytical, Almelo, Netherlands). For the WD-XRF analysis, fused beads were prepared by an Eagon 2 from Panalytical. The obtained percentages in weight are shown in Table 1.

The final theoretical chemical composition of the obtained hydraulic binders is also shown in Table 1.

The raw mixtures were compressed and placed in a platinum crucible, following the preparation procedure presented by Santos et al. [10], consisting in heating the mixture to a temperature, which will be called maximum temperature, stabilizing it for 30 minutes, quenching the melt by extracting the crucible from the oven, and finally, leaving it to cool down to air exposure. In order to obtain different percentages of crystalline pseudowollastonite, the binder C/S 1.1 and C/S 1.25, were heated at different maximum temperatures, ranging from 1485 °C to 1550 °C. After cooling, the amorphous hydraulic binders were mechanically removed from the crucibles and were ground in a ringmill for 180 s with propanol, obtaining a particle size below 35 μm. The ground powder was then dried at 50 °C in a stove for about 1 h in order to remove the propanol. X-Ray Diffraction (XRD) analysis verified that the grinding process did not introduce any detectable changes of mineralogy. Pastes were prepared by mixing the amorphous powder with water at a water/binder weight ratio of 0.375. The pastes were poured into proper molds with dimensions of 20 × 20 × 40 mm^3^ and cured in a moisture-controlled environment with a relative humidity over 95%.

Hydration and chemical evolution were tracked, and compressive strength measurements were performed at the ages of 7, 28, and 90 days, thus that a relationship between hydration product development and mechanical properties could be established over time. 

Table 2 shows the designation of each lot of samples, which includes the respective ratio C/S and the maximum temperature at which the material equilibrated, always within the wollastonite + liquid domain.

The phase development during hydration was tracked by X-Ray Diffraction (XRD, X’Pert Pro, Malvern Panalytical, Almelo, Netherlands)-Rietveld analysis, at 7, 28, and 90 days of hydration. The acquisition of the XRD diffractogram was performed with an X’Pert Pro diffractometer using monochromatic CuKα1 radiation (*λ* = 1.54059 Å) and working in reflection geometry (*θ*/2*θ*). The optics configuration was a fixed incident anti-scatter slit (1°), a fixed divergence slit (1/2°), a fixed diffracted anti-scatter slit (1/2°), and an X’Celerator detector, working in scanning mode with maximum active length. For each sample, data were collected from 5° to 70° (2*θ*). The samples were rotated during data collection at 16 rpm, and the X-ray tube was maintained at 45 kV and 40 mA. Rietveld analysis was performed using the Panalytical software Highscore Plus. To enable the quantification of the amorphous content, 20% in weight of an internal standard of corundum (99.9% α-Al_2_O_3_ from Alfa Aesar, Haverhill, MA, United States) was added to each mixture. 

The isothermal calorimetry analysis was performed immediately after the binder, and the water was mixed (w/c 0.375) through a TAM Air Instruments—Waters Sverige AB (Sollentuna, Sweden). The hydro-reactive binders’ characteristic exothermic heat flow was measured using glass ampoules, with Milli Q water as a reference. All experiments were performed at 20 °C, and the heat of hydration data was collected for 7 days.

Compressive strength tests were performed on paste prisms with dimensions of 20 × 20 × 40 mm^3^ at 7, 28, and 90 days of hydration. The compressive strength tests were performed on the prism samples using an Ibertest Autotest 400/10 equipment by applying a force rate of 2.4 kN/s.

Thermogravimetric analysis (TGA) measurements (ELTRA GMBH, Haan, Germany) were performed in order to assess the amount of water contained in the structure of the hydrated products at 7, 28, and 90 days of hydration. The ELTRA multichannel TGA device was used following the temperature program developed by the Central Laboratory of CIMPOR-Cimentos de Portugal (Lisbon, Portugal). Tests were run at constant heating rates between fixed temperatures (105 °C, 250 °C, 500 °C, and 950 °C), each temperature was maintained until a constant mass was achieved. In the first step, between the room temperature and the 105 °C a heating rate of 4 °C/min was applied, from 105 °C to 250 °C, a heating rate of 10 °C/min, in the last two steps, from 250 °C to 500 °C and between 500 °C and 950 °C, the applied heating rate was 15 °C/min.

In this paper, only the TGA data collected during the range of 105 °C to 500 °C were analyzed. This choice was made based on the fact that the entirety of the C–S–H bound water was lost in this temperature range [13,14], and thus assuming, when the samples achieved their mass constancy at 500 °C, that, by the end of the test, all the C–S–H were dehydrated.

Low-temperature differential scanning calorimetry (LT-DSC) measurements were performed on samples at 90 days of hydration under a nitrogen atmosphere to investigate the degree of hydration of the samples by characterizing the water confined in the hydrated paste. The samples were weighed and placed into a sealed aluminum pan (diameter 5 mm, capacity 25 mL, from NETZSCH, Selb, Germany). The device used was a DSC 200F3 Maia model from NETZSCH (Selb, Germany), and the data were analyzed with Proteus^®^ 8.6 software. The temperature program used in this work was based on reference [15]: Equilibrate at 20 °C for 10 minutes; cool from 20 °C to −80 °C at 2 °C/min; equilibrate at −80 °C for 10 minutes; heat from −80 °C to 20 °C at 2 °C /min. 

The fractured surface of samples with 28 days of hydration was observed through Scanning electron microscopy (SEM) using a FEG-SEM JEOL 7001F equipment (JEOL Ltd., Tokyo, Japan) at a tension of 15 kV. All samples were covered with a gold deposition before the SEM analysis. 

## 3. Results

### 3.1. XRD/Rietveld

**Figure 1:**
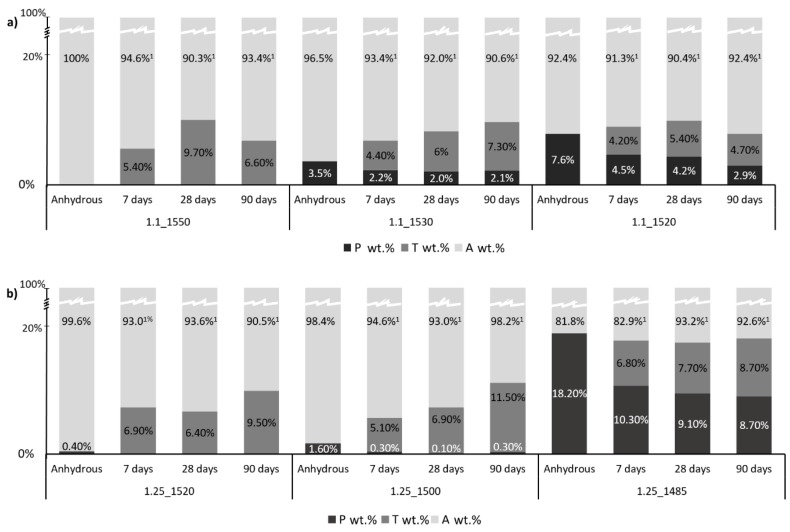
Rietveld analysis of samples C/S 1.1 (**a**) and C/S 1.25 (**b**) prepared at different clinkering temperatures, the percentages in weight of different phases (Note: P-Pseudowollastonite; T-Tobermorite, A-Amorphous; ^1^ The amorphous phase wt.% includes the anhydrous and the C–S–H) is shown.

Figure 1a,b show, respectively, the phase composition in weight percentage of the anhydrous hydraulic binder and the correspondent pastes of samples C/S 1.1 and C/S 1.25, at different hydration ages: 7, 28, and 90 days. Data were obtained by Rietveld analysis.

It can be noted, for both samples C/S_1.1 and C/S_1.25, as the hydration time increased, the content of hydration products, namely tobermorite structures, also increased. For anhydrous samples, the quantity of pseudowollastonite increased with the decrease of the maximum temperature, however, in the case of C/S 1.25, getting different percentages of initial pseudowollastonite by decreasing the maximum temperature, happened to be difficult due to the proximity of the ratio C/S 1.25 to the eutectic point in the CaO-SiO_2_ equilibrium phase diagram. Samples of C/S 1.25 seemed to form more crystalline hydration products than samples of C/S 1.1. The paste of sample C/S_0.9 (with initial phase composition of 0.9 wt.% of Pseudowollastonite and 99.1 wt.% of amorphous phase) was not analyzed at 7, 28, and 90 days because it did not react when in contact with water.

### 3.2. Isothermal Calorimetry

**Figure 2:**
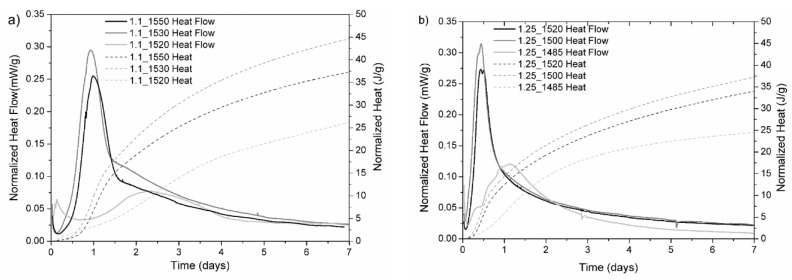
Heat flow and cumulative heat (normalized to the mass of binder powder) as a function of time of hydration: (**a**) C/S 1.1 Samples; (**b**) C/S 1.25 Samples.

Figure 2 shows the normalized heat flow versus time evolution, respectively, for samples C/S 1.1 (Figure 2a) and C/S 1.25 (Figure 2b). The respective cumulative release was also shown as a dashed line. The data were normalized to the mass of anhydrous binder powder.

Sample C/S_1.1_1530 and 1.1_1550 (Figure 2a) reached the peak maximum at around 19 and 23 h of hydration, respectively. In the case of sample 1.25_1520 and 1.25_1500 (Figure 2b), the maximum heat release was registered at 10 h of hydration. The samples 1.1_1520 and 1.25_1485, prepared at the lowest temperature, presented a weak and late exothermic peak. It can be observed, though, that there was a tendency which linked the decrease of the intensity peak and the delay of the hydration reaction with the increase of pseudowollastonite presence (decrease of maximum temperature). The peak intensities for samples 1.1_1550 and 1.25_1520, which were both brought to the liquid phase, were similar and were lower compared to the peak intensities of samples 1.1_1530 and 1.25_1500, which were heated just below the liquid transition temperature. There seems to exist an optimized condition for sample 1.1_1530 (~3.5 wt.% of initial pseudowollastonite), which reached a peak of 0.32 mW/g at 19 h of hydration. Sample C/S_0.9 did not present any exothermic peak and, therefore, was not reported.

### 3.3. Compressive Strength

**Figure 3:**
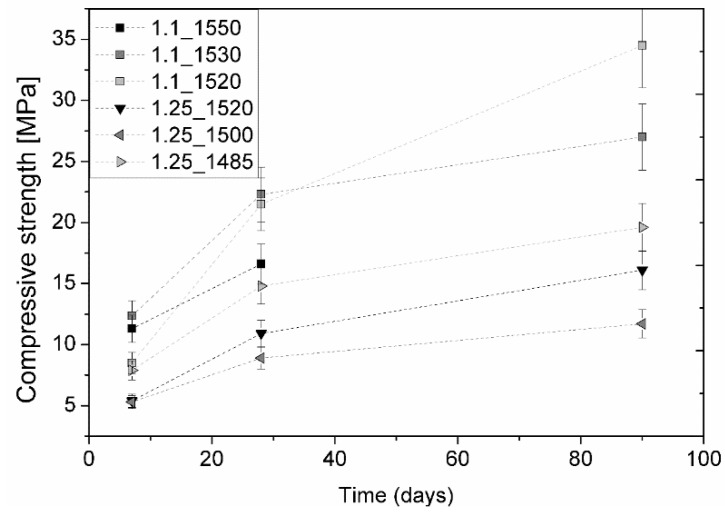
Compressive strength development of the pastes produced from samples C/S_1.1 and C/S_1.25. Note: water/binder = 0.375 in weight. (The error bars correspond to the maximum error associated with measurements).

Figure 3 shows the compressive strength evolution for the group of samples C/S_1.1 and C/S_1.25 at 7, 28, and 90 days of hydration. Sample 0.9 did not set, therefore, this group of samples were discarded in the analysis. 

At the early ages, namely, until seven days of hydration, the presence of pseudowollastonite did not seem to be relevant for the compressive strength performance, with the obtained mechanical resistances similar between each other. Samples C/S_1.1 present, overall, significantly higher compressive strength at 28 and 90 days of hydration than samples of C/S_1.25. In both CS_1.1 and CS_1.25, there was an increase in compressive strength with a decrease of the maximum temperature during the preparation process, which corresponded to the increase of the pseudowollastonite percentage. Sample 1.1_1520, initially with 7.6 wt.% of pseudowollastonite (represented as a light grey square in Figure 3), presented an impressive evolution of compressive strength between 7 and 90 days by increasing from 8.5 MPa to 34.5 MPa. The strength of sample 1.1_1550 at 90 days was not measured due to experimental constraints. Samples C/S_1.25 had a significantly lower compressive strength, about half when compared to samples C/S_1.1. In the case of C/S_1.25, the results obtained at seven days of hydration were consistent with the results obtained for advanced ages, with sample 1.25_1485 being the samples with better compressive strength.

### 3.4. Thermogravimetry Analysis (TGA)

**Figure 4:**
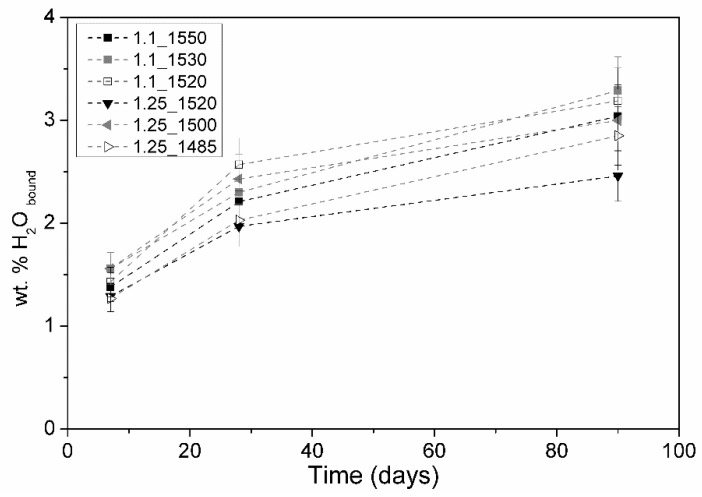
Evolution of the percentage of water incorporated in the structure of pastes produced as a function of hydration age. (The error bars correspond to the maximum error associated with measurements).

Figure 4 shows the TGA results. As expected, all tested samples showed an increase in the structural water along with the hydration time.

At 90 days of hydration, overall, samples C/S_1.1 presented values of structural water higher than those of samples C/S_1.25. The samples brought to the highest maximum temperature during preparation, 1.1_1550 and 1.25_1520, presented the lowest relative structural water values when compared to the respective samples produced at lower maximum temperatures, which means that the presence of pseudowollastonite accelerated the hydration mechanism.

### 3.5. Scanning Electron Microscopy (SEM)

**Figure 5:**
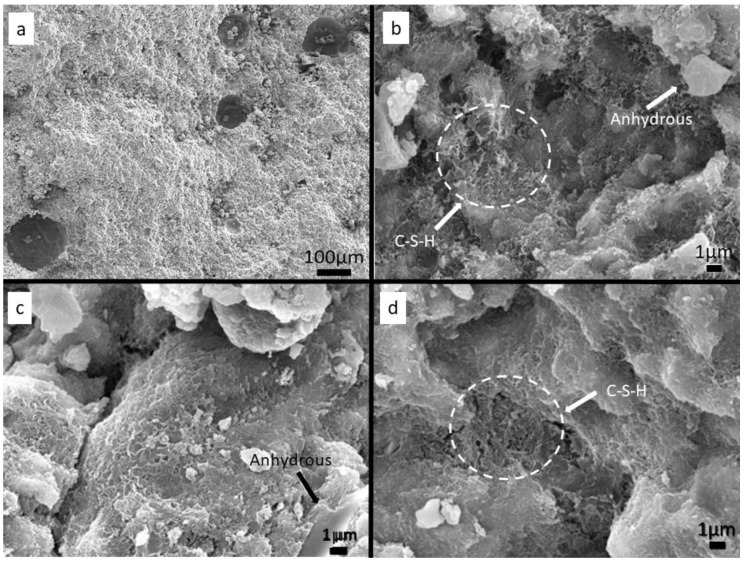
SEM images of samples C/S 1.1 with 28 days of hydration: (**a**) 1.1_1550 at a magnification of 100×; (**b**) 1.1_1550 at a magnification of 5000×; (**c**) 1.1_1530 at a magnification of 5000×; (**d**) 1.1_1520 at a magnification of 5000×.

Samples 1.1_1550, 1.1_1530, and 1.1_1520 at 28 days of hydration were observed through SEM analysis. Figure 5a shows, as an example, the image of a fractured area of sample 1.1_1550 at 100× of magnification. Various pores can be observed whose diameters ranged from a few µm to ~200 µm. This type of porous morphology was confirmed in each sample. Figure 5b–d show the morphology at 5000× magnification of samples 1.1_1550, 1.1_1530, and 1.1_1520, respectively. In every sample, the presence of short fibers and lamellar structures, typical of hydrated calcium silicate phases, can be observed (examples of C–S–H structures have been highlighted in Figure 5). In some cases, few agglomerates of anhydrous material, which did not react, were spotted (see highlights in Figure 5).

The structures observed in samples C/S 1.25 were equivalent to the ones of samples C/S 1.1 and were not reported.

### 3.6. Low-Temperature Differential Scanning Calorimetry (LT-DSC)

**Figure 6:**
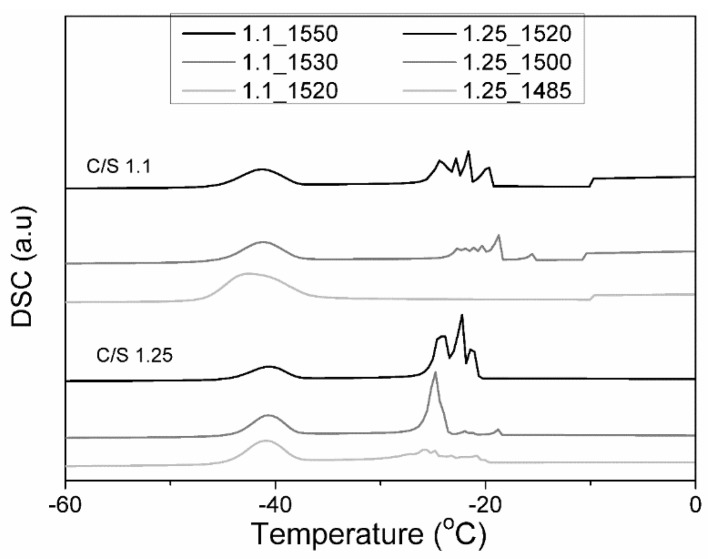
Differential scanning calorimetry (DSC) cooling thermograms of the set of samples C/S 1.1 and C/S 1.25 at 90 days of hydration.

According to the Jennings colloidal model II [16], which describes the C–S–H microstructure, water inside cement paste can be located in interlamellar space (dimension <1 nm), small gel pores (SGP), with dimensions 1–3 nm; and in the large gel pores (LGP),with dimensions 3–12 nm. Free water confined in these pores will freeze at different temperatures according to the pore’s dimension [15,17]. As hydration takes place, water can only be only located in the SGP. By using LT-DSC, the water location was investigated. Figure 6 shows the thermograms of cooling scans of the group of samples under study at 90 days of hydration. Freezable water was evident as heat flow from the sample, during cooling, was translated as a peak in the thermogram, whose temperature was related to the pore size where the water was contained [17]. Two types of peaks can be observed: One between −20 °C and −30 °C, which corresponded to the water confined in the LGP space [15], and the other below −35 °C, which corresponded to the water confined in the SGP [15]. Looking at the thermograms, it can be observed that most of the samples, with the exception of sample 1.1_1520, presented peaks corresponding both to the water confined in the LGP and the water confined in the SGP, which indicated that the hydration process was incomplete [15,16,17]. In the case of sample 1.1_1520, the thermogram suggested that hydration was completed and that the only water presented was located in the SGP. It can also be noted that with an increase in the pseudowollastonite content, the intensity of the peaks above −35 °C increased, while the intensities of the peaks at −20 °C decreased, indicating that the presence of pseudowollastonite accelerated the hydration mechanism, confirming the TGA results.

## 4. Discussion

R.L Santos et al. [12], Hoshino et al. [18], and Kazuhiro et al. [19] found a linear relationship between the quantity of formed C–S–H and the compressive strength results. This trend was confirmed by the results reported.

The percentage in weight of the calcium silicate hydrates (C–S–H) present in the pastes at 7, 28, and 90 days of hydration, was calculated using the amount of bound water determined by TGA and by assuming the models presented in the works of Qomi et al. [20] and Richardson [21], which compute correlations between the water to silicon molar ratio (H/S) and the C/S molar. The following Equation (1) was given in the work of Richardson [21] to define the water to silica molar ratio (H/S) as a function of the C/S molar ratio in the C–S–H products with a tobermorite-like structure and valid for conditions of severely dried samples:

(1) $$\frac{H_{2}O}{\mathrm{Si}}=\frac{19\cdot \frac{\mathrm{Ca}}{\mathrm{Si}}-7}{17},$$

Equation (2):

(2) $$\mathrm{wt}.{\%C}_{x}{\mathrm{SH}}_{y}=\left(\frac{\frac{{\mathrm{wt}\%\mathrm{SiO}}_{2\mathrm{reacted}}}{\left(\frac{{\mathrm{wt}\%\mathrm{SiO}}_{2\mathrm{initial}}}{100}\right)}+{\mathrm{wt}\%H}_{2}O_{\mathrm{bound}}}{100+{\mathrm{wt}\%H}_{2}O_{\mathrm{bound}}}\right)\times 100,$$

where,

(3) $$\mathrm{wt}.{\%\mathrm{SiO}}_{2\mathrm{reacted}}=\frac{{\mathrm{wt}\%H}_{2}O_{\mathrm{bound}}}{\left(\frac{W}{S}\right)\times \frac{M\left(H_{2}O\right)}{M\left({\mathrm{SiO}}_{2}\right)}},$$

and the value $\mathrm{wt}.{\%\mathrm{SiO}}_{2\mathrm{initial}}$ is taken from Table 1, was then used to calculate the % of C–S–H produced upon hydration of the pastes prepared, by assuming that:

(i) All the bound water, as determined by TGA, is removed in the temperature range between 105 °C and 500 °C;

(ii) The C–S–H produced during the hydration of these pastes present a C/S molar ratio similar to the precursor materials.

**Figure 7:**
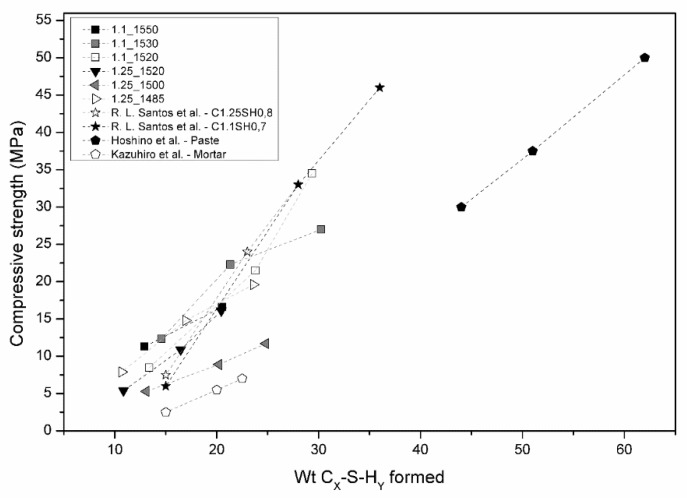
The plot of weight percentage of C–S–H formed versus the obtained compressive strength results in this work. In addition, plotted are other results of R. L. Santos et al. [10], Hoshino et al. [18]. and Kazuhiro et al. [19].

Figure 7 plots together data taken from the literature and the experimental data obtained in the present work, confirming the linear relationship where higher values of bound water provide higher amounts of hydration products and, consequently, higher compressive strengths results.

A linear relationship between compressive strength and cumulative heat release in cement and mortar materials was found by Bentz et al. and other researchers [22,23]. However, in the present study, this relationship was not observed. Even though samples 1.25_1520 and 1.25_1500 were the samples that released the largest heat of hydration (see Figure 2), their compressive strength showed lower values than samples 1.1_1550 and 1.1_1530 (Figure 3).

Comparing the evolution of the compressive strength results at 7, 28, and 90 days of hydration of the samples under study (Figure 3), with the percentage of initial pseudowollastonite phase shown in Figure 1a,b. It can be observed that at seven days of hydration, in the case of sample C/S 1.1, the presence of pseudowollastonite does not seem to be advantageous as concerns mechanical strength. In addition, samples 1.1_1530 and 1.1_1520 show an increase in compressive strength along time with the increase of the percentage of the initial pseudowollastonite phase. Furthermore, sample 1.1_1520, which presented very low strength at seven days of hydration, grow to 34.5 MPa at 90 days, representing the best result amongst the samples under study. 

The compressive strength values of C/S 1.1 samples are considerably better than samples 1.25, even in the totally amorphous samples (1.1_1550 and 1.25_1520), which confirms that the “quality” of the C–S–H of samples C/S 1.1 is better, as was previously reported by R.L. Santos et al. [8]. Sample 1.25_1485, which has around 19 wt.% of the initial pseudowollastonite, which presented the best results within the C/S 1.25 samples set at all of the hydration ages. From these results, it is evident that the presence of initial pseudowollastonite in the amorphous composition increases the compressive strength.

**Figure 8:**
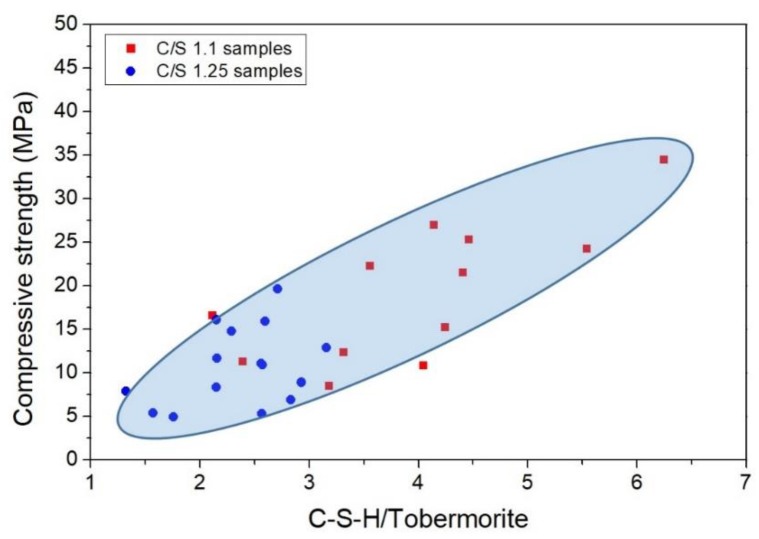
Relationship between the compressive strength results and the C–S–H/Tobermorite weight ratio of samples C/S 1.1 and C/S 1.25.

Figure 8 shows the relationship between compressive strength and the C–S–H/tobermorite weight ratio. The values used for tobermorite were obtained through XRD analysis (See Figure 1a,b), while the values of the wt.% of CSH were calculated through Equation (2). The C/S 1.1 samples present higher C–S–H/tobermorite ratio and higher compressive strength results compared to C/S 1.25 samples. 

This study suggests that, in these cases, the main contributor to the mechanical properties is the amorphous C–S–H structure, which seems to work as a “glue” involving the tobermorite nanocrystals, suggesting that without the presence of the C–S–H, tobermorite nanocrystals do not improve in a relevant manner of the mechanical properties of the clinker paste. 

The low calcium binders investigated in the present work presented low cumulative heat of hydration (see Figure 2a,b), about one order of magnitude lower than the cumulative heat of hydration of an ordinary PC cement e.g., [24], e.g., Type I PC conforming to the American Society for Testing and Materials (ASTM) C 150 (Standard Specification for Portland Cement) at two days of hydration, which shows approximately 250 J/g of binder [25] versus the 20–25 J/g cumulative heat released by the samples shown here (see insets in Figure 2a,b). This is mainly due to the fact that these new binders did not contain any C_3_S/phase, which constituted between 50% and 80% of the PC [26] and was the main contributor to the formation of C–S–H [26,27] and, consequently, to heat release. In the case under study, besides the absence of C_3_S, we did not observe the presence of calcium aluminate phases and the formation of portlandite, which would further contribute to the heat release of PC. All these facts suggest that we are dealing with different mechanisms of hydration and C–S–H formation, than for PC, and that these mechanisms are still unclear and require further research. The decrease in the heat release may represent not only a critical key to prevent or minimize the evolution of cracks in large constructions [28], but also an improvement of later-age concrete strengths and durability performance. However, the low calcium amorphous binders presented in this work are far behind the expected strength, and further studies and adjustment of the paste preparation protocol will be done in order to improve the mechanical properties at early and later ages.

The SEM observation (Figure 5) reveals the presence of large pores, which suggest the presence of air bubbles during the paste setting, associated with an excess of water or with the lack of pastes compaction during sample preparation. Pores decrease the elastic modulus and, consequently, have an important impact on the strength and durability of the cement [29]. Moreover, through the SEM analysis, it was possible to identify a structure that is described in the literature as a typical C–S–H gel-type structure [30,31,32]. 

## 5. Conclusions

In this work, three C/S ratios have been investigated C/S 0.9, C/S 1.1, and C/S 1.25, by testing different temperatures of production, and it was concluded that among the three C/S molar ratios tested, the sample with the ratio C/S 0.9 did not present any hydraulic reactivity until 90 days of hydration, while the sample C/S 1.1 presented the best mechanical performances.

Using XRD analysis, an increase in the percentage of endogenous crystalline pseudowollastonite, and a decrease in the clinkering temperature was observed. The percentage of crystalline pseudowollastonite phase varied between 0 wt.%–7.6 wt.% and 0 wt.%–18 wt.%, respectively, in the case of C/S 1.1 and C/S 1.25 samples. The minimum temperature tested was 1520 °C and 1485 °C, respectively, for the C/S_1.1 and C/S_1.25, the authors do not infer anything for temperatures lower than these. 

Compressive strength tests were performed on each sample at 7, 28, and 90 days, and the results were compared to the typical values of PC. As expected, the samples with higher hydration times presented higher structural water contents, more hydration products (C–S–H), and, consequently, higher compressive strengths. Samples C/S 1.1 and 1.25 achieved 34.5 MPa (1.1_1520, 7.6 wt.% of initial pseudowollastonite) and 19.6 MPa (1.25_1485, 8.7 wt.% of initial pseudowollastonite) at 90 hydration days, respectively, against the 15 MPa and 11 MPa of their respective totally amorphous samples at the same age of hydration. However, these values are still lower than for PC, whose typical value at the same age is 58 MPa. The authors believe that more work can be done and that these binders have to be further investigated, for example, avoiding the sample porosity by improving the paste preparation protocol.

Isothermal calorimeter analysis showed to be an accurate tool for reactivity prediction, as the presence of heat of hydration indicates the existence of binder reactivity. The samples presented in this work showed a release of heat of hydration an order of magnitude lower than for PC. This promising result suggests the potential of these low calcium binders in the field of large mass concrete construction, such as in the case of dams and large raft foundation. 

## Figures and Tables

**Table 1:** Raw material composition and raw-mix combinations of the amorphous hydraulic binders with C/S molar ratios of 0.9, 1.1, and 1.25. The respective theoretical compositions of the amorphous material produced are presented in the bottom rows.

C/S Molar Ratio	1.1	1.25	0.9	Composition (wt.%)									
**Raw Materials**	**Wt.%**			**SiO_2_**	**Al_2_O_3_**	**Fe_2_O_3_**	**CaO**	**MgO**	**SO_3_**	**K_2_O**	**Na_2_O**	**TiO_2_**	**P_2_O_5_**
Limestone	63.78	66.57	58.97	0.20	0.16	0.14	99.11	0.30	0.04	0.02	0.04	0.02	-
Sand	34.97	32.2	39.74	96.94	1.29	0.16	-	0.02	-	0.52	0.11	-	-
FCC	1.25	1.23	1.29	39.48	51.39	0.52	0.13	0.17	0.09	0.02	0.48	0.81	0.22
**Binder**	**Wt.%**			**SiO_2_**	**Al_2_O_3_**	**Fe_2_O_3_**	**CaO**	**MgO**	**SO_3_**	**K_2_O**	**Na_2_O**	**TiO_2_**	**P_2_O_5_**
C/S 1.1	100	-	-	47.87	1.59	0.16	49.26	0.16	0.02	0.26	0.08	0.02	0.00
C/S 1.25	-	100	-	44.97	1.56	0.16	52.39	0.17	0.02	0.25	0.08	0.02	0.00
C/S 0.9	-	-	100	52.86	1.65	0.16	44.34	0.15	0.02	0.29	0.08	0.02	0.00

**Table 2:** Sample nomenclature.

Sample	C/S	Maximum Temperature (°C)
0.9_1550	0.9	1550
1.1_1550	1.1	1550
1.1_1530	1.1	1530
1.1_1520	1.1	1520
1.25_1520	1.25	1520
1.25_1500	1.25	1500
1.25_1485	1.25	1485
